# Exonuclease Ribozyme Encoded in a Circular Genome: On the Initiation of the RNA World

**DOI:** 10.3390/life16081287

**Published:** 2026-08-04

**Authors:** Minglun Liang, Chunwu Yu, Hongyu Jiang, Wentao Ma

**Affiliations:** 1 Hubei Key Laboratory of Cell Homeostasis, College of Life Sciences, Wuhan University, Wuhan 430072, China; 2 College of Computer Sciences, Wuhan University, Wuhan 430072, China

**Keywords:** the origin of life, early evolution, the RNA world, computer simulation

## Abstract

The “RNA World” hypothesis posits a reasonable scenario for the origin of life. A question central to this scenario is: which RNA species may have emerged as the first ribozyme, thereby initiating the earliest stage of life? To date, experimental and computational studies have focused on ribozymes playing a constructive role in RNA synthesis—such as those facilitating template-directed copying or nucleotide synthesis. However, the answer remains unconvincing, primarily because their functional complexity likely demands lengthy sequences, making de novo emergence improbable. Here, we propose an alternative scenario: a destructive ribozyme, which is potentially quite short, may have emerged first. This ribozyme could cleave other RNA molecules to generate building blocks for its own replication. Notably, the dilemma for a destructive ribozyme is that it may degrade molecules of its own type, thus making it difficult to thrive as an RNA species. Using computational modeling, we demonstrate that an exonuclease ribozyme encoded within a circular RNA genome (thus evading self-attack) can spread within an RNA pool and may subsequently give rise to those constructive ribozymes. Our findings offer a new perspective on the initiation of the RNA world, thereby enriching both theoretical and experimental frameworks for understanding the origin of life.

## 1. Introduction

The origin of life remains one of the most profound unsolved questions in the natural sciences. In this field, the “RNA world” is a leading hypothesis: it proposes that RNA acted as both the genetic material and a functional molecule in a primordial stage of life [1,2,3]. The hypothesis is grounded in three logical points: (1) “capable of undergoing Darwinian evolution” represents the critical feature of a life form; (2) heredity and functionality are two indispensable prerequisites for Darwinian evolution to occur; (3) and it is improbable that two distinct classes of polymers—one carrying heredity and the other carrying functionality (with an “information linkage” between them, like DNA and proteins in modern organisms)—could have emerged simultaneously at the start. Indeed, as we know, RNA serves as the genetic material in viroids and a portion of viruses. The discovery of ribozymes promoted the formal proposal of the “RNA world” hypothesis [1,4,5]. A subsequent finding—that the catalytic core of the ribosome is RNA [6,7]—proved to be the most compelling evidence supporting the notion that “proteins were invented by RNA”. Coupled with many other lines of evidence, the “RNA world” hypothesis is now widely accepted and commonly serves as a “model scenario” for both experimental and theoretical investigations in the field [8,9,10,11].

On account of the difficulties involved in prebiotic RNA synthesis, there were proposals suggesting the potential existence of pre-RNA worlds—involving RNA-like polymers, which were subsequently taken over by RNA [2,8]. Later, owing to advances in the area of prebiotic synthesis, especially studies concerning nucleotide synthesis [12,13,14,15,16], it seems much more feasible that the RNA world emerged de novo. Then, the question becomes clearer: how could the RNA world have started from a prebiotic pool [17]?

Notably, based on the logic that “the simpler, the more likely to have emerged de novo”, it has been hypothesized that a “naked” stage existed at the onset of the RNA world [18,19,20,21,22]. In other words, it is initially RNA molecules themselves that acted as the units of Darwinian evolution (i.e., Darwinian entities), and RNA-based “protocells” emerged later—marking the “first major transition” in evolutionary history [18,23,24]. In this context, the question of how the RNA world started is in fact: which RNA species, as a Darwinian entity, may have emerged first in a prebiotic nucleotide pool—that is, representing the most primordial ribozyme?

An RNA species capable of catalyzing template-directed RNA synthesis—and thus replication (commonly referred to as an “RNA replicase”, here REP for short)—is a long-proposed candidate for the earliest ribozyme to emerge in the RNA world [2,8,17,25,26]. For three decades, sustained experimental efforts by RNA engineering, with in vitro evolution as a core approach, have been devoted to “constructing” an RNA polymerase ribozyme analogous to the modern RNA polymerase enzymes (i.e., using mononucleotides as substrates) [27,28,29,30,31,32,33,34,35,36]. As a key advance, a polymerase ribozyme was evolved in a water–ice medium to catalyze the copying of specific RNA templates of a length comparable to its own (~200 nt) [31]. However, its structural complexity precludes self-templating, and its large size gives rise to the potentially severe problem of “strand separation” during replication. Notably, some recent efforts have shifted to developing a polymerase that uses trinucleotides as substrates (namely, a “triplet polymerase ribozyme”—essentially a template-directed ligase) [37,38,39,40], which at least partially alleviates these two limitations (by unraveling ribozyme secondary structure and trapping dissociated RNA strands, respectively). However, like other engineered polymerase ribozymes, the triplet polymerase ribozyme also derives from the class I ligase ribozyme (which is already large) [27], thus remaining oversized (existing as a 135 + 153 nt heterodimer) [37]. A critical unsolved problem is in fact that the spontaneous emergence in the initial nucleotide pool of such a large ribozyme seems implausible. In light of this, a recent study performed a de novo selection from a random sequence pool and identified a 45 nt template-directed ligase ribozyme (QT45), which can synthesize both its complementary strand using a random triplet pool and a copy of itself using defined substrates, marking a major step towards a plausible REP in the early RNA world [41].

Simultaneously, theoretical work regarding the evolutionary dynamics of REP in the RNA world has also been conducted. For instance, computer simulations demonstrated that RNA-like “replicators” could spread in a “naked” phase via replicase-like function by favoring their own replication through limited dispersal [42,43]. More concretely, our previous simulation study suggested that the first emerging RNA replicase may have been a simple, loosely binding template-directed ligase, which would readily dissociate from the template to act as a template for its own replication [44]. This simple template-directed ligase is hypothesized to utilize both nucleotides and various oligomers as substrates aligned on the template (the REP assumed in the present modeling study is also such a simple ligase; see below). Notably, the recently reported QT45 ribozyme closely resembles this hypothetical simple ligase—that ribozyme is engineered to use trinucleotide substrates, but longer oligomers may also be ligated on the template; additionally, dinucleotides and mononucleotides can be utilized as well, albeit with lower efficiency [41].

Another candidate for the first ribozyme to emerge in the RNA world is a ribozyme catalyzing the synthesis of nucleotides (a nucleotide-synthetase ribozyme, NR). In fact, the fundamental mechanism of RNA replication is merely template-directed synthesis. Therefore, the first ribozyme to emerge in the RNA world need not be an REP, especially considering the feasibility of non-enzymatic template-directed synthesis [45]. Indeed, evidence is accumulating showing that such non-enzymatic synthesis can be rather efficient [46,47,48,49]. By supplying the basic building blocks for RNA synthesis, an NR could also favor its own replication and thus spread within the prebiotic pool. This scenario has been supported by our computer modeling [50] (notably, there is also modeling work demonstrating the scenario of REP-NR co-spread [51,52]). Experimental studies have confirmed the chemical feasibility of RNA-catalyzed nucleotide synthesis [53,54,55]. In fact, an important rationale for proposing NR as the earliest ribozyme also relates to the sequence length: its substrates (i.e., nucleotide precursors) are far smaller than those of REP (i.e., the template–substrate complex), potentially enabling the ribozyme itself to be shorter in sequence [50].

However, even a simple REP or NR (e.g., ~40–50 nt) might still exceed the feasible length for spontaneous emergence from a prebiotic nucleotide pool, especially given that sequence space expands exponentially with increasing sequence length. In principle, the potential lower length limitation of REP and NR may stem from their “constructive” nature—such ribozymes require a minimum length to fold into a stable, functional structure and participate in their relevant synthetic reactions. For instance, an REP should carry several key functions: general template and substrate binding, ligation of adjacent substrates on the template, and the capability to undergo multiple turnovers of these two processes; an NR should be capable of binding different substrates (e.g., ribose and a nucleobase or other molecular moieties) simultaneously and catalyzing their condensation. Indeed, as depicted for this tiny candidate of REP: “The low tolerance for mutations and deletions of QT45 likely reflects a high density of functional residues in the QT ribozyme core required to maintain the multiple structural and functional requirements of a polymerase ribozyme in a small RNA motif” [41].

Actually, given the feasibility of non-enzymatic template-directed synthesis, the first emerging ribozyme may be any one that could favor its own replication [45]. For example, a “destructive” ribozyme may cleave other RNA molecules to supply building blocks for its own synthesis, thus being capable of spreading under Darwinian evolutionary principles. In fact, the smallest known natural ribozymes are destructive: specifically, the small self-cleaving ribozymes, which catalyze the cleavage of their own RNA strands [56,57,58]. Among these, the well-known hammerhead ribozyme can be as short as ~50 nt. Excluding its substrate segment (the target of self-cleavage), the essential catalytic segment of this ribozyme spans only ~25–30 nt [59]. Interestingly, a recent study identified an even smaller self-cleaving ribozyme, designated the “lantern” ribozyme, which can be as short as 31 nt, with a 15 nt essential catalytic segment [60].

Remarkably, it is now clear that small self-cleaving ribozymes are common genomic elements throughout the modern living world [56,57,58]. Furthermore, in vitro selection experiments targeting non-natural self-cleaving ribozymes have shown that initially random-sequenced RNA pools can become heavily enriched with diverse self-cleaving ribozymes [61,62]. Beyond small self-cleaving ribozymes, many other well-characterized ribozymes—including group I introns, group II introns, the RNA components of spliceosomes, and RNase P—also catalyze reactions involving phosphodiester bond cleavage [63,64]. In reality, with the sole exception of peptidyl transferase (in the ribosome), all other naturally occurring ribozymes catalyze RNA cleavage or related reactions [63,64]. Given the extreme conservation of the translation machinery, it is understandable that the catalytic role of RNA in ribosomal peptide synthesis cannot be substituted by proteins. By contrast, the widespread persistence of the ribozymes associated with RNA chain cleavage in modern organisms likely reflects a simple explanation: ribozymes are inherently suited to catalyzing such reactions.

One may then ask: is it plausible that a simple, destructive ribozyme emerged first in the RNA world? For instance, we may assume it is shorter than 20 nt. Such a ribozyme would be expected to cleave other RNA molecules to provide building blocks for its own replication. However, a critical question arises: a destructive ribozyme could also degrade RNA molecules of its own kind. How could such a ribozyme persist and undergo Darwinian evolution?

Relevantly, a recent simulation study conducted in our group suggests that circular genomes, which are widespread in the modern living world, may have their “root” in the RNA world [65]. Indeed, it has long been recognized that a linear polymer can undergo intramolecular cyclization once it reaches sufficient chain length, allowing its two termini to come into close proximity [66]. For RNA, such intramolecular end-to-end ligation is known to occur readily [67]. More notably, RNA products generated under prebiotically plausible conditions have been demonstrated to be enriched in “ring-like structures” [68]. In other words, circular RNAs were likely abundant in primordial RNA pools and could have participated in Darwinian evolution throughout the earliest stages of the RNA world [65].

In light of this, we propose that the simple, destructive ribozyme mentioned above may have been an exonuclease ribozyme (ER), which was encoded on a circular RNA—representing the first gene in the genome. In this manner, the gene could evade cleavage by the ribozyme. As for the ribozyme itself, we hypothesize that by folding into its functional conformation, it may avoid, at least to some extent, attack from other molecules of its own kind.

Therefore, the purpose of the current study is to model a scenario: could an ER encoded in a circular genome emerge from a nucleotide pool? If the answer is positive, could constructive ribozymes such as REP and NR emerge subsequently, thus potentially evolving towards a more complex RNA world? Certainly, the relevant mechanisms involved in these evolutionary processes are expected to be explored.

## 2. Results

### 2.1. About the Model

**Figure 1:**
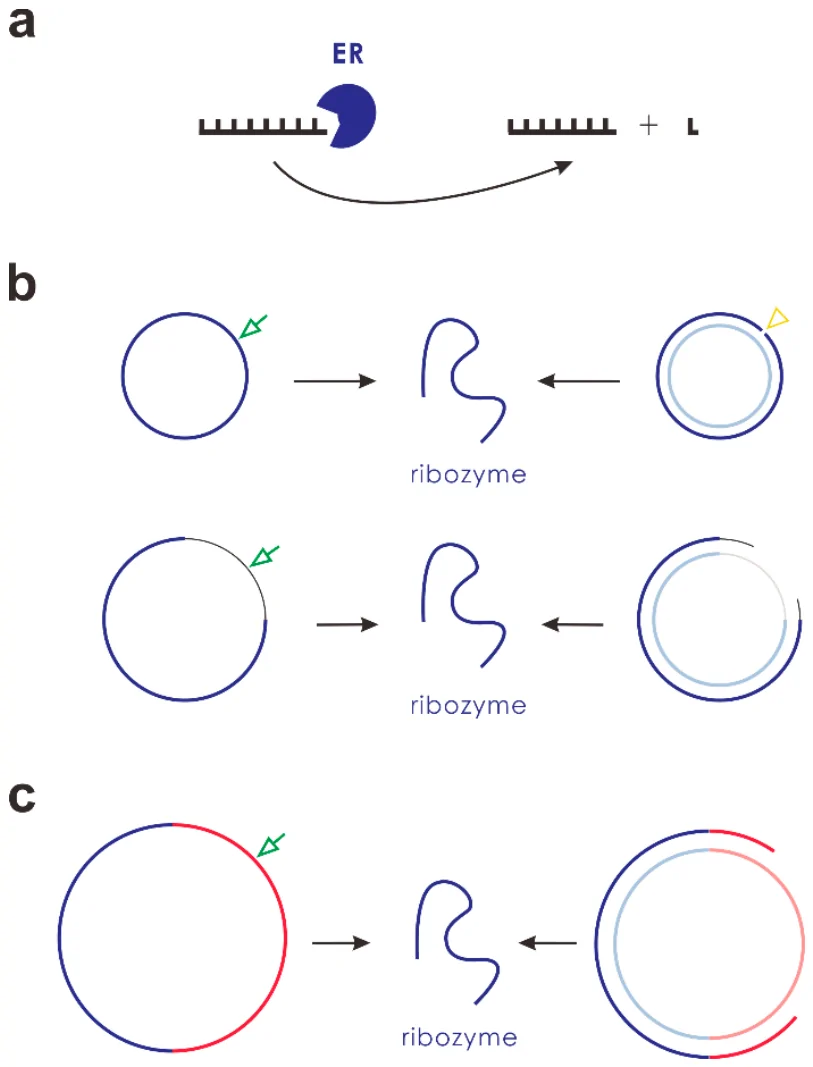
Conceptual schemes of the exonuclease ribozyme (ER) and circular genomes at the early stage of the RNA world. (**a**) An ER cleaves an RNA molecule at its chain end and releases a mononucleotide. (**b**) The upper panel corresponds to a “compact” single-gene genome. Blue lines represent the ribozyme sequence, and light-blue lines represent its complementary sequence. The ribozyme must be generated through precise breaking (the arrow) of the sense chain or through “accurate RNA synthesis” on the antisense chain template—specifically, initiation at the correct position and timely dissociation of the linear sense chain prior to circularization (the triangle). The lower panel corresponds to a single-gene genome containing a non-coding sequence. Thin black lines represent the non-coding sequence, and thin gray lines represent its complement. In this case, the ribozyme can be generated by any chain breaking event within the non-coding region (the arrow), or by dissociation of the linear sense chain that already contains the full ribozyme sequence from the template before complete circularization. (**c**) A two-gene genome. Red lines represent the sequence of the second ribozyme, and light-red lines represent its complement. A ribozyme may be generated by breaking the circular sense chain at the region of the other ribozyme (the arrow), or via dissociation of a linear sense chain carrying the full ribozyme sequence from the template.

We conducted computer simulations using a Monte Carlo model similar to those employed in our previous work concerning the naked stage of the RNA world [44,50,65,69,70], particularly the most recent one that accounts for circular RNA [65]. The system consists of a two-dimensional *N* × *N* square grid (with a toroidal topology to avoid edge effects). We adopt a two-dimensional system partly for simplicity and partly to reflect prebiotic milieus such as mineral surfaces (with dispersal limitation) for the naked stage, as supported by many studies [71,72,73,74]. Molecules, including nucleotide precursors, nucleotides, and RNA, are distributed within the grid rooms. In each time step (Monte Carlo step), relevant events may occur to the molecules with defined probabilities:

- Nucleotide precursors may be converted into nucleotides (randomly as A, G, C, or U).
- Nucleotides may assemble into linear RNAs through random ligation.
- A linear RNA may circularize via end-to-end ligation.
- Both linear RNAs and circular RNAs may undergo template-directed replication using nucleotides and oligomers as substrates.
- A circular RNA may turn into a linear one via the breaking of a certain phosphodiester bond.
- A linear RNA may also break into smaller fragments.
- A nucleotide may degrade back into a nucleotide precursor.
- A terminal nucleotide residue on a linear RNA may also degrade to a nucleotide precursor.
- A linear RNA molecule containing a characteristic sequence (domain) is hypothesized to exert a specialized function (i.e., it may act as a ribozyme): specifically, an ER cleaves other RNA molecules at their single-stranded termini (Figure 1a); an REP catalyzes the template-directed ligation of RNA; and an NR mediates the synthesis of nucleotides from their precursors. It should be noted that the defined domains are purely modeling abstractions that capture ribozyme sequence specificity. Accordingly, the terms “ribozyme”, “genome”, and “gene” used throughout this work do not correspond to real biochemically characterized molecular equivalents.
- Molecules may also move to an adjacent grid room.

For descriptions of the associated parameters, refer to Table 1; detailed explanations are provided in Section 4.

At the beginning of a simulation, a certain number of nucleotide precursors are introduced into the system. As time progresses, nucleotides and RNA will emerge. Conversely, the degradation of RNA and nucleotides may regenerate nucleotide precursors. Overall, the total amount of materials in the system remains constant. RNA sequences, which can replicate via template-directed synthesis, compete for these limited materials. Potentially, an RNA “species” with a specific sequence may spread (i.e., become thriving) in the system by virtue of its function, which favors its own replication.

Notably, the model has an “information resolution” at the nucleotide level, thus inherently making it suitable for studying early Darwinian evolution, which relies essentially on the sequence–function connection of RNA. In practice, the characteristic sequence of a functional RNA species (ribozyme) is arbitrarily presumed due to the lack of empirical examples, but this does not matter. The significant point is merely: if a characteristic sequence corresponds to a special function, can the species with this sequence prevail in “natural selection”?

### 2.2. The Spread of Exonuclease Ribozyme

**Figure 2:**
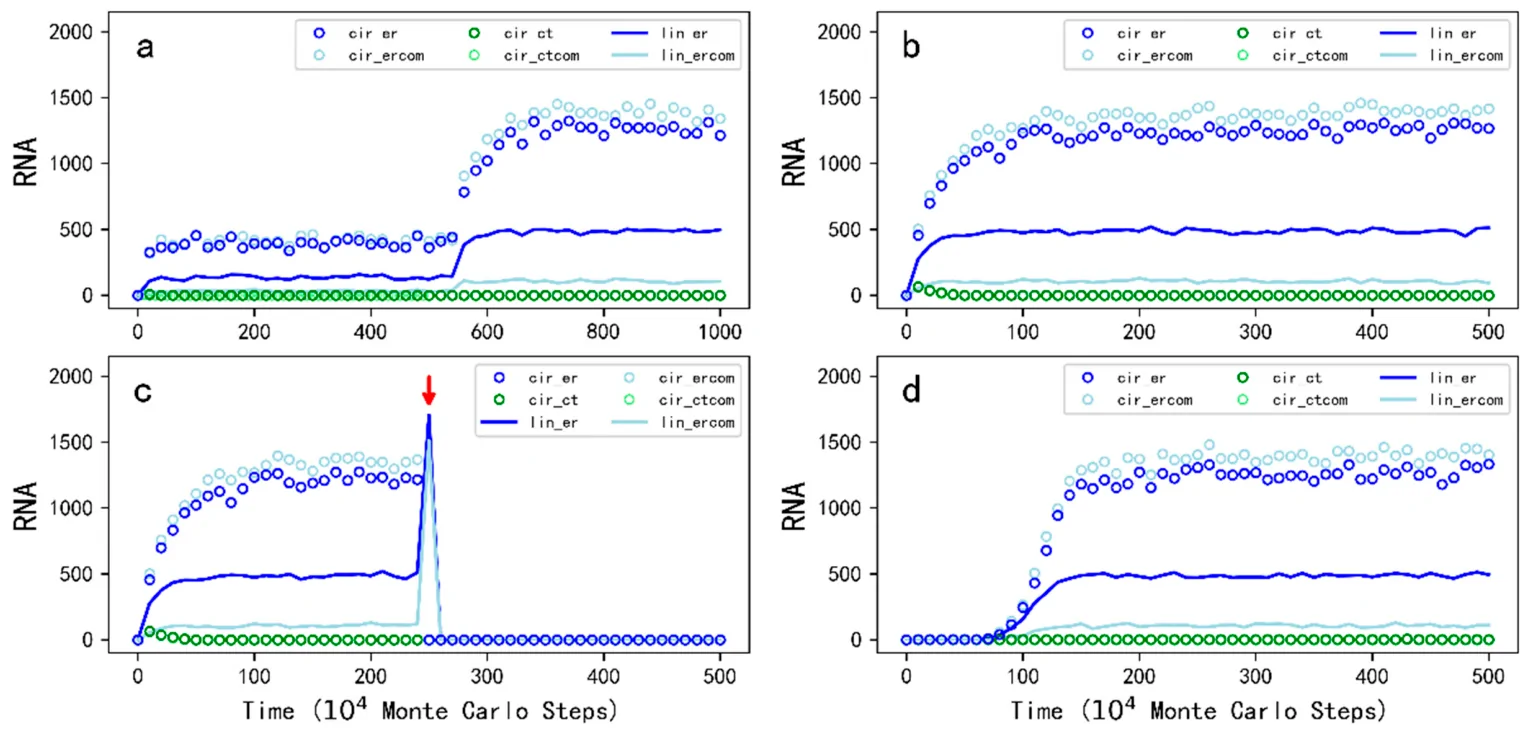
The spread of ER encoded in a circular genome. The vertical axis denotes the number of distinct RNA molecule types, as described in the legend. Legends: cir_er—circular RNA containing the ER sequence (i.e., the characteristic sequence of ER); cir_ercom—circular RNA containing the complement of the ER sequence; lin_er—linear RNA containing the ER sequence; lin_ercom—linear RNA containing the complement of the ER sequence; cir_ct—circular RNA containing the control sequence; cir_ctcom—circular RNA containing the complement of the control sequence. (**a**) At step 1 × 10^4^, 10 circular RNA molecules with the ER sequence (8 nt in length; see Table 1) and an equal number of circular RNA molecules with the control sequence (8 nt; see Table 1) are inoculated into the system (at randomly chosen locations; the same applies to all inoculations described below). (**b**) At step 1 × 10^4^, 10 circular RNA molecules with the ER sequence plus a 3 nt non-coding sequence (11 nt in total length) and an equal number of circular RNA molecules with the control sequence plus the non-coding sequence (11 nt) are inoculated into the system. (**c**) Based on the case depicted in (**b**), at step 2.5 × 10^6^ (the red arrow), all circular RNA molecules are artificially cleaved into linear forms—wherein the ER sequence and its complementary sequence are retained (i.e., cleavage sites are located within the non-coding region); *P_EL_* is set to 0; *F_LT_* is set to 1. (**d**) At step 1 × 10^4^, 20 circular RNA molecules (11 nt) with random sequences are inoculated into the system. One circular ER molecule (11 nt; identical to the one depicted in (**b**)) and one circular control molecule (11 nt) are inoculated into the system every 1 × 10^5^ steps.

In our model system, the exonuclease ribozyme (ER) exerts its catalytic function by cleaving the termini of other RNA molecules, thereby releasing free mononucleotides (Figure 1a). These monomers may be reused for the replication of ER itself, thereby enabling ER to spread (proliferate) throughout the system. However, this creates an inherent dilemma: an ER may also cleave other ER molecules or their complementary strands, thereby hindering its spread. As noted in the Introduction, we hypothesized that if the ribozyme is encoded within a circular genome, this dilemma would be resolved, thus enabling ER to propagate as a molecular species. This expectation was supported by our simulations. In the case depicted in Figure 2a, at 1 × 10^4^ Monte Carlo steps, 10 circular RNA molecules with the characteristic sequence of ER (8 nt in length; see Table 1) were inoculated into the system, alongside an equal quantity of control sequences. As a result, ER increased rapidly in the system (blue circles for circular ER sequences and the blue line for linear ER sequences), whereas the control species did not. Notably, linear ER sequences can function as ribozymes in the system.

Then, at approximately step 5.5 × 10^6^, the ER abundance (both linear and circular sequences) increased markedly (Figure 2a). This is because a compact circular genome is inefficient at generating a linear ribozyme, a process that requires precise strand cleavage or accurate template “transcription” (Figure 1b). Therefore, when a “non-coding sequence” was inserted into the circular genome by chance, the expanded genome outcompeted the compact ER genome and rose to dominance (Figure S1a). By “working in a more efficient way”, the ER genome subsequently spread to a higher level. The chance event of genome enlargement may occur via intermolecular ligation of a linear ER sequence and an unrelated linear fragment (associated with the parameter *P_RL_* in the model), followed by circularization of the resulting longer linear sequence (associated with *P_EL_*).

For the case shown in Figure 2a, the expanded genome is 11 nt in length, containing a 3 nt non-coding sequence (Figure S1a). In fact, our simulations revealed that for a ribozyme with an 8 nt characteristic sequence, evolution toward an 11 nt genome was the most common outcome observed, although other lengths (e.g., 9 nt and 10 nt) were also observed (Figure S1b–d). This phenomenon is highly analogous to our relevant findings regarding REP and NR, in which we first proposed the plausibility of circular genomes in the RNA world [65]. To generate a robust platform (i.e., thriving at a higher level renders populations less susceptible to random fluctuations) for subsequent analysis, we chose to initially inoculate an 11 nt expanded circular genome (rather than the 8 nt compact genome), thereby directly achieving stable propagation of ER at a high level (Figure 2b).

Notably, in both the case shown in Figure 2a and that shown in Figure 2b, we observed quantitative asymmetry between ER and its complementary sequence: linear ER is significantly greater in number than its linear complement, while circular ER is somewhat more abundant than its circular complement. At first glance, this phenomenon is somewhat unexpected because each strand acts as a replication template for its complement. For example, in our relevant investigation regarding REP and NR, no such asymmetry exists [65] (refer to Figure S2).

First, we reasoned that the disparity in linear chains arises because ERs are partially protected from cleavage by folding (*P_RP_* = 0.5; see Section 4), whereas their complements are not. However, abolishing this protection midway (*P_RP_* = 0 at step 2 × 10^6^) had little effect (Figure S3a). We then hypothesized that the key driver is the fact that “an ER molecule cleaves other molecules” per se. Indeed, granting ER cleavage activity to ER complements minimized this imbalance (Figure S3b). When combining two adjustments, the difference disappeared (Figure S3c), supporting the conclusion that these two factors jointly drive the observed asymmetry. Notably, when disparity between linear ER and its complement vanishes, the disparity between circular ER and its complement also disappears (Figure S3c). We deduced that the higher copy number of the circular ER complement stems from the abundance of linear ER: given their similar sequence information, the circular form shares oligonucleotide substrates with the linear form; therefore, RNA fragments dissociated from the linear ER template may be recruited by the circular ER template, thereby promoting the synthesis of circular ER complements. Indeed, shutting down linear RNA template activity midway (*F_LT_* = 0; see Section 4) immediately abolished disparity between circular ER and its complement (Figure S3d). Just after the shutdown (step 2 × 10^6^), circular RNAs (ER and its complement) increased because only they can act as template. However, without the contribution of linear templates, linear ER was significantly reduced. That is, functional ribozyme concentrations declined, and the abundance of the circular genome fell accordingly (Figure S3d). This indicates that the origin system (before disabling the linear templates) is actually characterized by a combined linear–circular genome, consistent with a relevant conclusion of our previous work on a circular REP genome [65]: a “purer” circular genome likely emerged later in the RNA world, with a distinct genome-ribozyme division of labor.

We then turned to a key mechanism we hypothesized: is the circular genome, which shields the ER gene from attack by the ER ribozyme, really important for the spread of ER as an RNA species? Based on the case depicted in Figure 2b, at step 2 × 10^6^, all circular RNA molecules were “artificially broken” into linear molecules (wherein circular ER was converted into linear ER, and the same applied to their complementary sequences). Meanwhile, the system was adjusted such that RNA circularization was no longer permitted (*P_EL_* = 0), and linear RNA molecules acted as templates as efficiently as circular RNAs had previously (*F_LT_* = 1). As a result, the levels of linear ER and its complementary sequences, after a temporary increase (due to the “artificial breaking” of their circular counterparts), decreased sharply (Figure 2c). In other words, ER cannot propagate when the circular genome form is “banned”, even if the linear form is assumed to have no shortcoming with respect to template capability.

**Figure 3:**
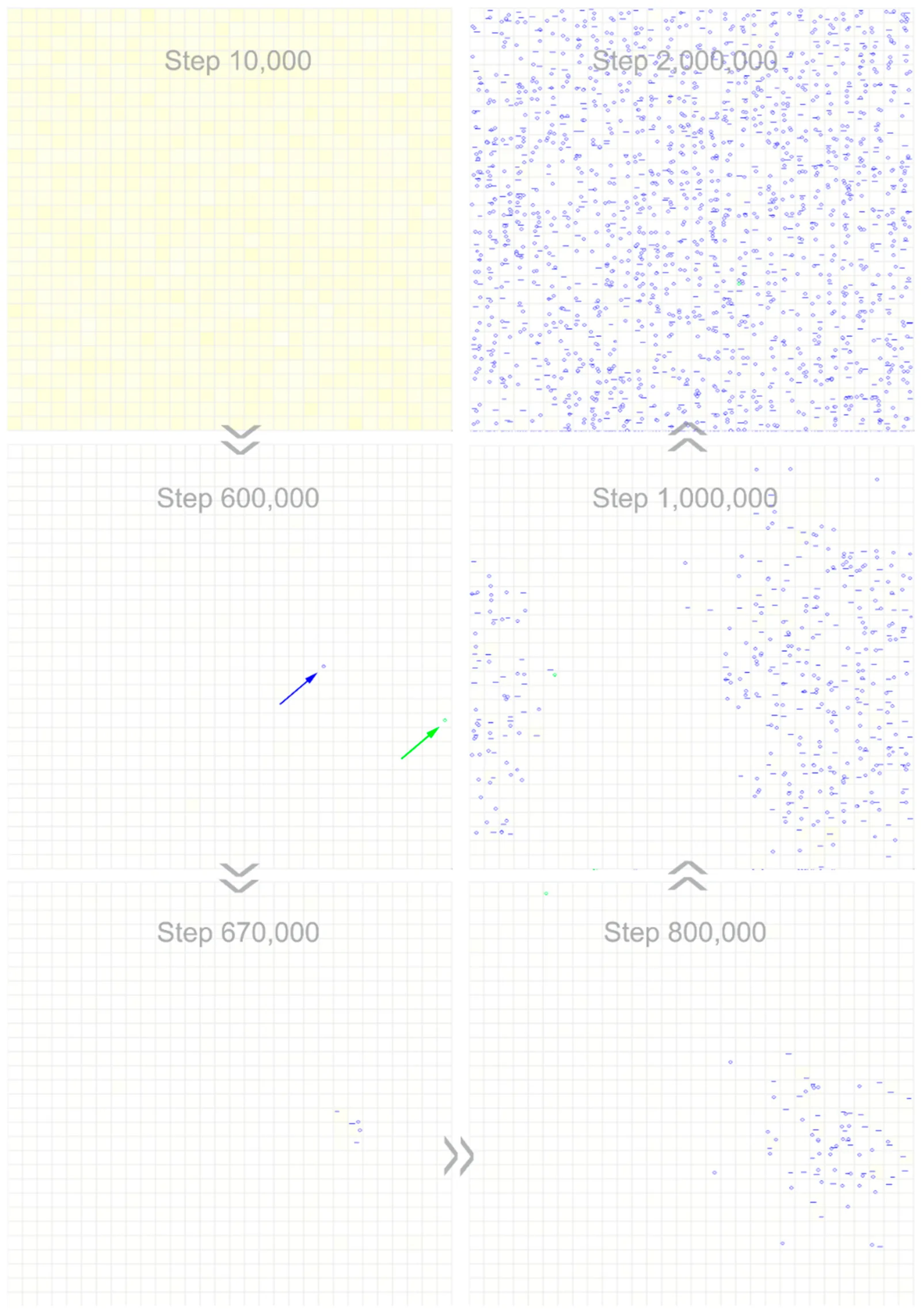
Snapshots illustrating the spread of ER encoded in a circular genome. The evolutionary dynamics of this case are depicted in Figure 2d. Raw materials (nucleotide precursors) are represented by a yellow background, where the color depth indicates their quantity in the corresponding room (gray-lined grid). Circular molecules are denoted by circles (with sizes correlated), while linear molecules are denoted by line segments (with lengths correlated). Blue corresponds to ER, and green corresponds to the control. The snapshot at step 10,000 represents the system before the inoculation of the 20 random-sequence RNA molecules—raw materials are abundant at this stage. The inoculation of one ER molecule every 100,000 steps is performed when the availability of raw materials is significantly lower. This situation can be observed in the snapshot at step 600,000, which captures the key inoculation event that subsequently drives the spread of ER (the blue arrow points to the inoculated ER molecule, and the green arrow points to the inoculated control molecule). The subsequent snapshots demonstrate the gradual spread of ER throughout the system. Note that the grid has a toroidal topology.

Similar to our previous modeling studies [44,50,70], to avoid the influence of stochastic RNA degradation events in the early stage, we inoculated multiple (10) circular ER molecules to investigate the corresponding evolutionary dynamics (Figure 2a,b). The observed spread of ER already suggests that this RNA species could have emerged de novo from the postulated nucleotide pool. In reality, the simultaneous appearance of these specific RNA molecules is highly implausible. However, a single ER molecule may have appeared repeatedly over the extended timescale of life’s origin. As illustrated in Figure 2d, we simulated one such scenario: one circular ER molecule (11 nt in length, consistent with Figure 2b) and one control molecule were inoculated into the system every 1 × 10^5^ steps, and ER eventually propagated throughout the system. Importantly, to eliminate any first-mover bias (where early species benefit from high initial concentrations of raw materials, i.e., nucleotide precursors), 20 circular RNA molecules with random sequences (also 11 nt) were introduced into the system prior to the periodic inoculation of ER. As evident from the snapshots (Figure 3), at step 6 × 10^5^, when the critical inoculation event triggering the subsequent spread of ER occurs, the concentration of nucleotide precursors was significantly lower than that in the initial pool (step 1 × 10^4^). By step 6.7 × 10^5^, both circular ER (circles) and linear ER (line segments) have already proliferated; at step 8 × 10^5^, they began to spread locally. Subsequently, at step 1 × 10^6^, their expansion became extensive (note that the model system possesses a toroidal topology). Ultimately, at step 2 × 10^6^, ER molecules dominated the full simulation grid.

### 2.3. About the Influence of Parameters

**Figure 4:**
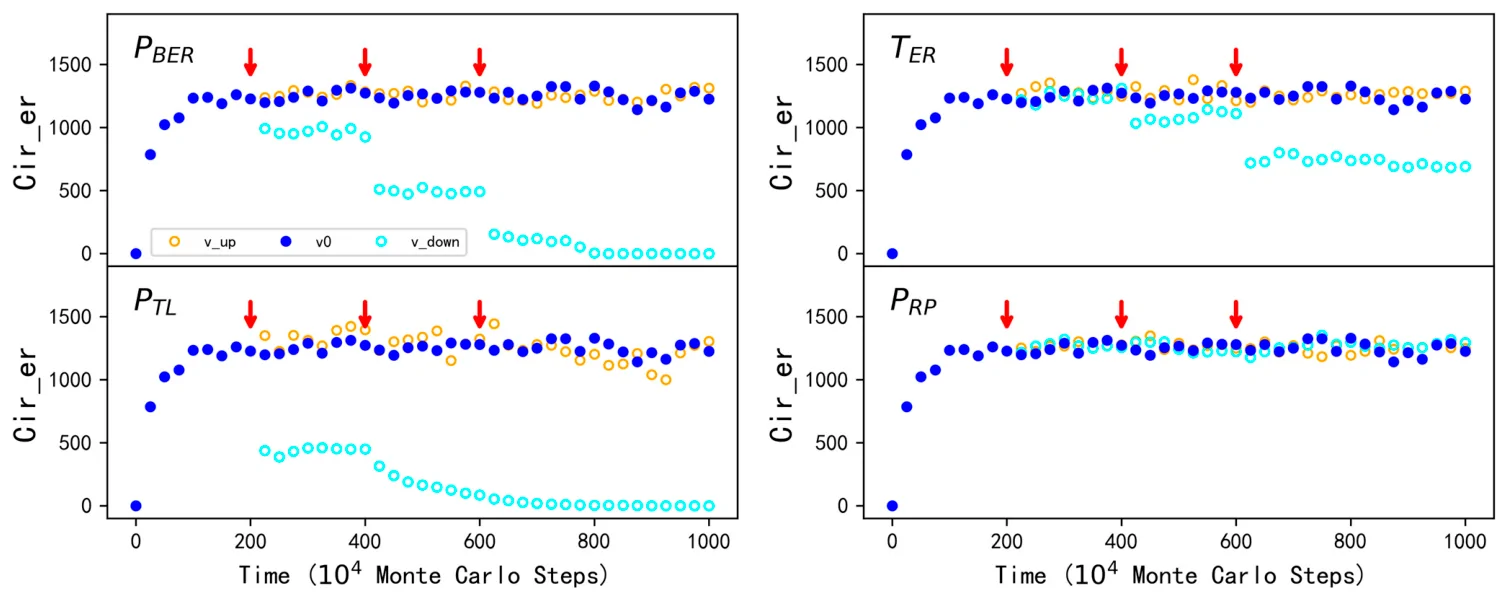
The influence of several key parameters on the spread of ER. The vertical axis denotes the number of circular RNA molecules containing the ER sequence, serving as an indication of the level of ER spread. Legends: v0—all parameters are set to default values; v_up—the relevant parameter depicted in the top-left corner of the panel is turned up (other parameters remain at their default values); v_down—the relevant parameter is turned down (the legends apply to all subfigures). Red arrows mark the critical steps at which parameter adjustments are performed. For (*P_BER_*), the default value of 0.9 is turned up to 0.95, 0.98 and 0.99 at these respective points, and turned down to 0.2, 0.05 and 0 at these points. For (*T_ER_*), the default value of 10 is turned up to 20, 50 and 100, and turned down to 5, 2 and 1. For (*P_TL_*), the default value of 0.05 is turned up to 0.1, 0.2 and 0.5, and turned down to 0.02, 0.01 and 0.005. For (*P_RP_*), the default value of 0.5 is turned up to 0.8, 0.9 and 0.95, and turned down to 0.2, 0.1 and 0.

Firstly, to confirm that the spread of ER is attributable to the function of the ribozyme, we examined the influence of *P_BER_* (the probability of phosphodiester bond breaking catalyzed by ER; see Table 1). Indeed, as ribozyme function was gradually reduced, the spread of ER was attenuated, and when the probability was finally set to 0, the spread was completely suppressed (Figure 4-*P_BER_*). Another parameter associated with the efficiency of ER is *T_ER_* (the number of times an ER acts within a single time step). With the stepwise reduction in this parameter, the spread of ER was also weakened (Figure 4-*T_ER_*). Next, given our hypothesis that ER first emerged in the RNA world (i.e., prior to the appearance of ribozymes such as REP), the replication of ER in the simulation relies on non-enzymatic template-directed synthesis of RNA (see Introduction). As anticipated, gradual reduction in *P_TL_* (the probability of template-directed ligation) suppressed the spread of ER and eventually eliminated it entirely (Figure 4-*P_TL_*). As noted earlier, the folding-dependent resistance of ERs to cleavage by other ERs appears to exert only negligible effects on the system (Figure S3a). Consistent with this observation, adjustments to *P_RP_* had little impact on the spread of ER (Figure 4-*P_RP_*). This finding is somewhat unexpected and highlights that, even though “ER molecules can attack one another”, ER may still spread as an RNA species—provided that “its gene is protected within a circular genome”.

The analysis of the remaining parameters is presented in Figure S4. Notably, the default parameter values we adopted are nearly optimal for the spread of ER (as illustrated in both Figure 4 and Figure S4): modifying these values—either increasing or decreasing them—could hardly improve the level of ER (see Box S1 for explanations of a few exceptions). This indicates that our parameter exploration was highly effective (see Section 4 for details of the “parameter exploration”). In addition, the spread of ER exhibits robustness against moderate parameter variations (the specific values used in Figure 4 are given in its legend, and those in Figure S4 are listed in Table S1). This result strengthens the plausibility of the ER-related scenario proposed in our modeling study.

### 2.4. RNA Chain Length and Topology Distribution: Destructive vs. Constructive

As mentioned above, we noticed a quantitative distinction between ER and its complementary sequence in the system (Figure 2), which was not observed during the spread of REP or NR (Figure S2). We then further analyzed the RNA chain length and topology distribution of the ER-spreading system, aiming to compare them with those of the systems involving these two constructive ribozymes.

**Figure 5:**
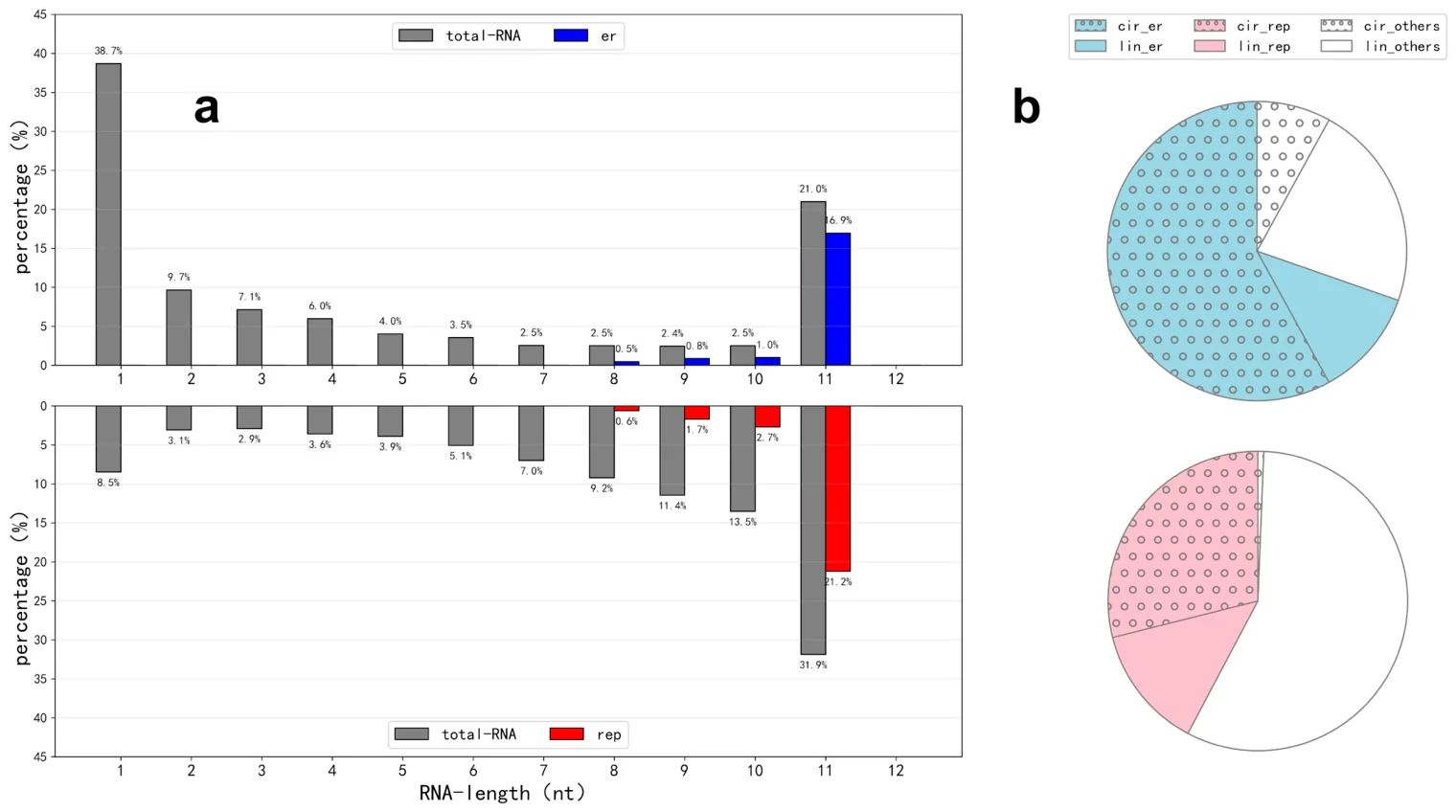
RNA distribution of the ER-spreading and REP-spreading systems. The charts depict the states of the two systems at step 5 × 10^6^, when the corresponding populations have reached stable spread (see Figure 2b and Figure S2a for their evolutionary dynamics, respectively). (**a**) The chain length distribution (the upper panel for the ER system and the lower panel for the REP system). The bars denote the percentage distribution of RNA molecules by quantity (there is no RNA longer than 11 nt). Legends: total-RNA—all RNA molecules; er—RNA molecules containing ER or its complementary sequence; rep—RNA molecules containing REP or its complementary sequence. (**b**) The topology distribution (the upper panel for the ER system and the lower panel for the REP system). The pies illustrate the proportion of total material (i.e., mass calculated based on nucleotide count) within the 8–11 nt range (wherein ribozymes are distributed). Legends: cir_er—circular RNA containing ER or its complementary sequence; lin_er—linear RNA containing ER or its complementary sequence; cir_rep—circular RNA containing REP or its complementary sequence; lin_rep—linear RNA containing REP or its complementary sequence; cir_others—circular RNA neither containing the ribozyme sequence nor its complementary sequence; lin_others—linear RNA neither containing the ribozyme sequence nor its complementary sequence.

Again, there is an obvious difference in chain length distribution between the ER-spreading system and the REP-spreading system (Figure 5a). The former contains significantly more mononucleotides than the latter, which can be attributed to the function of ER—cleaving RNA molecules at their termini. Additionally, it can be observed that, from 2mers to 10mers, the ER system generally exhibits a descending trend, whereas the REP system shows an ascending trend. This difference should have arisen from the respective destructive and constructive natures of the two ribozymes. In any case, it implies that, in the former system, more short oligomeric substrates are available for template-directed synthesis, whereas, in the latter system, there may be more parasitic RNAs (long oligomers tending to act as templates) that interfere with ribozyme spreading.

Analysis of the topological distribution of 8mers to 11mers (representing the length range of a ribozyme in the model—the characteristic domain is 8 nt long, and an active ribozyme should be shorter than 1.5 times the domain length; see Section 4) revealed that RNA sequences lacking the ribozyme-characteristic domain (i.e., parasites; the white area in the pie charts) are significantly more abundant in the REP system—with the linear fraction (the empty white area) being particularly prevalent (Figure 5b). In fact, for a single-gene circular genome, even if a non-coding sequence exists, many “false breaking or synthesizing” events are still likely to occur (e.g., breaks occurring within the ribozyme-characteristic domain; refer to Figure 1b), thereby generating these linear parasites. Interestingly, in the ER system, the linear parasites can be largely degraded by the ribozyme. As a related observation, circular parasites (the white area with circles) are significantly more abundant in the ER system than in the REP system because only by adopting a circular form can parasitic RNAs evade cleavage by ER.

As another case of a constructive ribozyme, NR exhibits a spreading pattern similar to that of REP, both in terms of RNA chain length and topology distribution (Figure S5). In addition, to provide another instance of a destructive ribozyme, we modeled a modified version of ER, which cleaves the ends of RNA molecules randomly by 1, 2 or 3 nt in length (experimental evidence for such random cleavage by ribozymes exists [75,76,77]). This ER variant, termed “ER13” here, can also spread in the system (Figure S6a), and this spreading is indeed attributable to its catalytic function (Figure S6b). The spreading pattern of ER13 is highly similar to that of the original ER, except that 2mers and 3mers are more abundant and 1mers are less abundant in the system (Figure S5)—a result consistent with our expectations.

### 2.5. The Subsequent Emergence of Constructive Ribozymes

If ER emerged as the first ribozyme in the RNA world, other ribozymes—for example, constructive ribozymes such as REP and NR, as previously proposed in the field—should have arisen subsequently. We therefore investigated whether this scenario could be recapitulated in our model system. Notably, for a multi-gene circular genome, a non-coding sequence is no longer required for efficient ribozyme production [65] (see Figure 1c for an illustration of the situation concerning a two-gene circular genome).

**Figure 6:**
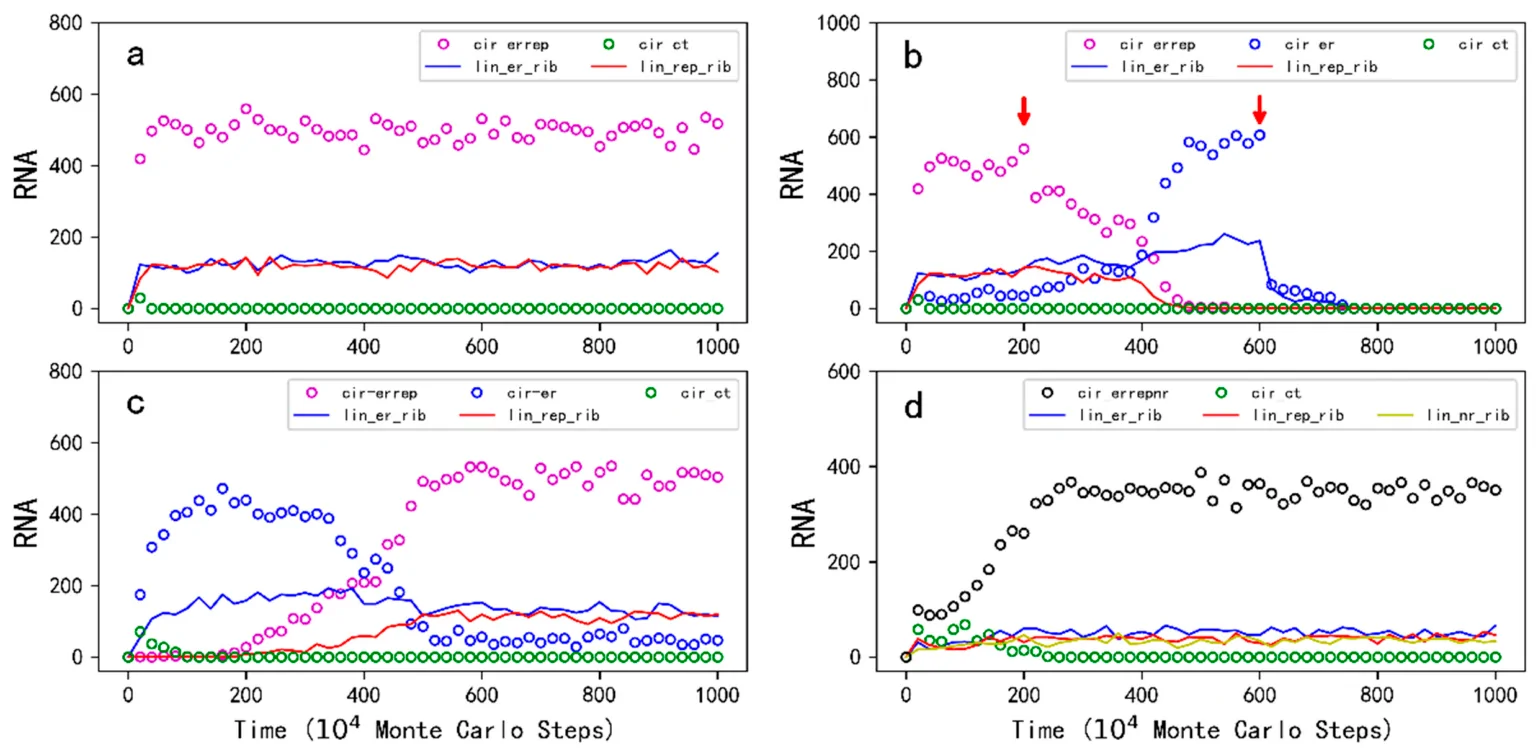
The spread of multi-gene genomes. Legends: cir_errep—circular RNA containing the ER and the REP sequence; cir_ct—circular RNA containing the control sequence; lin_er_rib—linear RNA containing the ER sequence that functions as a ribozyme (i.e., an active ribozyme is assumed to be less than 1.5 times the length of its characteristic sequence; see Section 4 for explanation); lin_rep_rib—linear RNA containing the REP sequence that functions as a ribozyme; cir_er—circular RNA containing the ER sequence (but no REP sequence); cir_errepnr—circular RNA containing the ER, the REP and the NR sequence; lin_nr_rib—linear RNA containing the NR sequence that functions as a ribozyme. (**a**) At step 1 × 10^4^, 10 circular RNA molecules with the sequences of ER and REP (16 nt in length, 8 nt for one gene), together with an equal number of control RNA molecules (also 16 nt in length), are inoculated into the system. (**b**) Based on the case shown in (**a**), *P_TLR_* is set to 0 (i.e., the catalytic ability of REP is turned off) at step 2 × 10^6^ (the left arrow), and *P_BER_* is set to 0 (i.e., the catalytic ability of ER is turned off) at step 6 × 10^6^ (the right arrow). (**c**) At step 1 × 10^4^, 10 circular RNA molecules with the ER sequence and a non-coding sequence (16 nt in length, 8 nt for each), together with an equal number of control RNA molecules (also 16 nt in length), are inoculated into the system. The non-coding sequence is assumed to be “GAGUCACU”, which differs by only one residue from the REP sequence (“GAGUCUCU”; see Table 1 for reference). (**d**) At step 1 × 10^4^, 10 circular RNA molecules with the sequences of ER, REP and NR (24 nt in length, 8 nt for each gene), together with an equal number of control RNA molecules (also 24 nt in length), are inoculated into the system.

**Figure 7:**
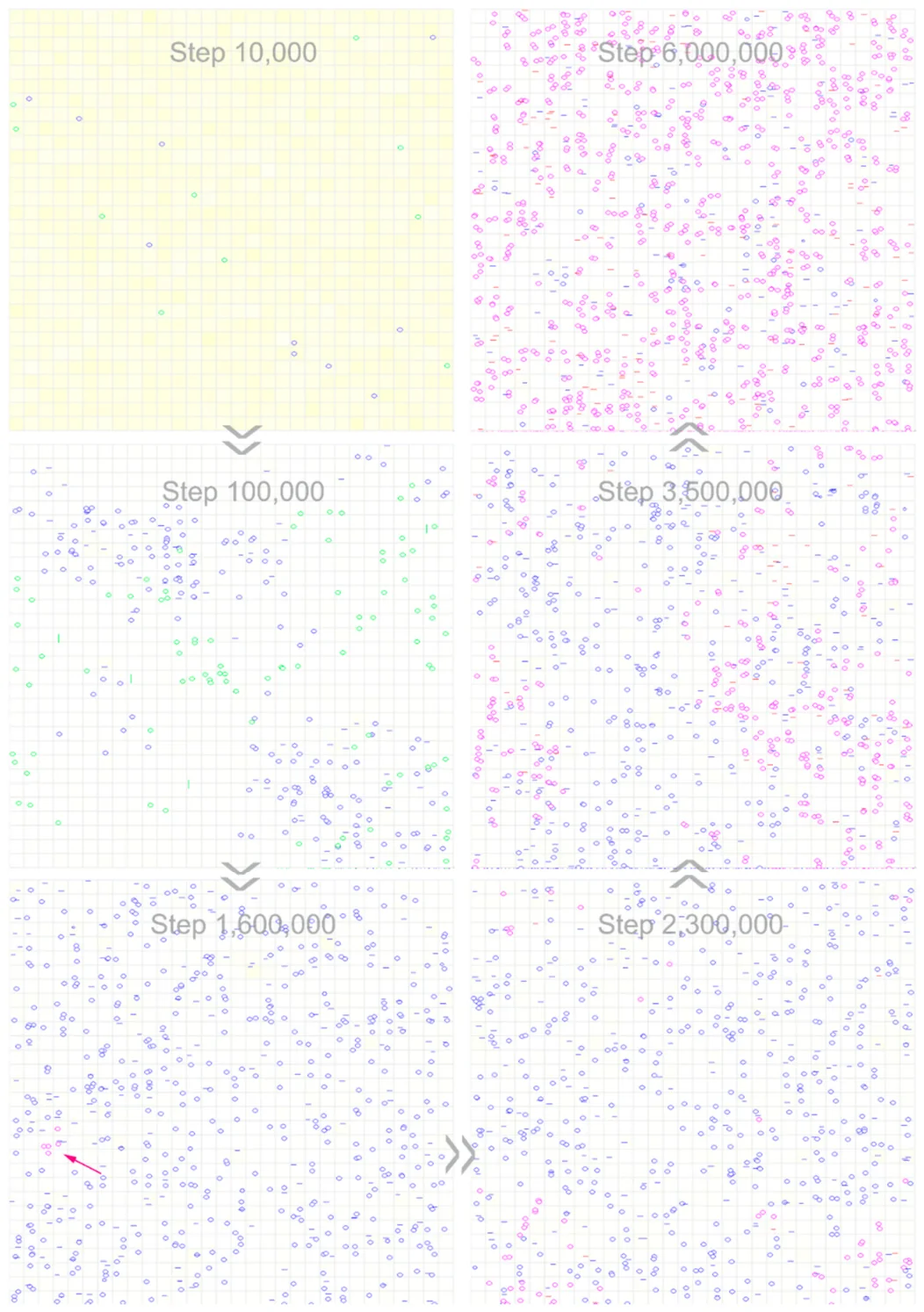
Snapshots illustrating the emergence of an ER-REP genome naturally from an ER genome-spreading system. The evolutionary dynamics of this case are depicted in Figure 6c. Raw materials (nucleotide precursors) are represented by a yellow background, where the color depth indicates their quantity in the corresponding room (gray-lined grid). Circular molecules are denoted by circles (with sizes correlated), while linear molecules are denoted by line segments (with lengths correlated). Blue circles denote circular RNA molecules containing the ER sequence (but no REP sequence). Green circles denote circular RNA molecules containing the control sequence. Blue line segments denote linear RNA molecules containing the ER sequence that function as ribozymes. Magenta circles denote circular RNA molecules containing both the ER and REP sequences. Red line segments denote linear RNA molecules containing the REP sequence that function as ribozymes. The snapshot at step 10,000 shows the inoculation of 10 circular ER molecules (containing a non-coding sequence) and 10 control RNA molecules. The snapshot at step 100,000 shows the spread of the circular ER genome. The snapshot at step 1,600,000 shows the appearance of circular RNA molecules containing both the ER and REP sequences (the arrow). The subsequent snapshots demonstrate the gradual spread of the circular ER-REP genome throughout the system and the simultaneous fading of the single-gene genome containing ER.

First, we inoculated a two-gene circular genome containing ER and REP and found that it can spread in the system (Figure 6a). When the function of REP was turned off midway, the two-gene genome faded away, leaving only the single-gene ER genome to spread (Figure 6b). This indicates that REP function indeed contributes to the propagation of the ER-REP genome. Certainly, subsequent inactivation of ER function collapsed the spread of the one-gene ER genome. We then inoculated a circular genome containing ER and a non-coding sequence to see whether an ER-REP genome could spread naturally via mutation of the non-coding sequence into REP (to facilitate the mutation, the non-coding sequence was assumed to differ from the REP sequence by a single nucleotide). Interestingly, such spontaneous emergence was observed in our simulation (Figure 6c; see Figure 7 for snapshots of the simulation case). Finally, it was revealed that the spread of a three-gene genome containing ER, REP, and NR is also plausible (Figure 6d), demonstrating the potential for further evolution.

## 3. Discussion

An NASA-formulated “working definition” of life for astrobiological exploration states: “Life is a self-sustaining chemical system capable of undergoing Darwinian evolution” [78,79,80]. The definition is widely accepted because it captures the two essential aspects of life: self-sustainment and the capacity to undergo Darwinian evolution [81]. However, these two aspects are so distinct that merging them within a single definition creates ambiguity. For instance, we can refer to a self-sustaining chemical system, but how can an individual system undergo Darwinian evolution? Darwinian evolution only operates on populations or genetic lineages. Therefore, the concept of life should be split—for example, by defining “living entity” and “life form” separately, with the former related to self-sustainment and the latter to Darwinian evolution [81]. For those who prefer a concise definition, it should at least be modified to: “Life is a sort of self-sustaining chemical system capable of undergoing Darwinian evolution” [11].

In the field of the origin of life, there has long been a debate surrounding the question of “metabolism first or replication first?” [82,83,84,85,86]. From the perspective of the conceptual analysis of life mentioned above, this debate is essentially: “self-sustainment first or Darwinian evolution first?”. In fact, the “self” of a modern living system can be regarded as being “defined” by its genetic information. Even if some self-sustaining chemical systems might have existed prior to the origin of Darwinian evolution, the meaning of “self” should have shifted after Darwinian evolution emerged, becoming intrinsically linked to the newly arisen genetic information [22]. In other words, the core logic underlying the origin of life lies in the origin of Darwinian evolution, followed by the subsequent emergence of self-sustainment as we know it (for the purpose of adaptation). Therefore, in the scenario of the RNA world, the emergence of the first ribozyme (or rather, the first gene) is a key event that initiated Darwinian evolution, thereby initiating the evolutionary trajectory that led to all life on Earth. The present paper focuses on this critical issue, which has puzzled researchers for decades since the proposal of the RNA world hypothesis.

We reasoned that a destructive ribozyme, i.e., the exonuclease ribozyme (ER), which might be much smaller than the previously proposed constructive ribozymes, such as the replicase ribozyme (REP) and nucleotide-synthetase ribozyme (NR), may have emerged first. The destructive ribozyme may cleave other RNA molecules to supply building blocks for its own replication. To avoid self-attack, the ER may have been “encoded” in a circular genome. Here, our computer modeling provides support for the plausibility of the relevant evolutionary dynamics (Figure 2a,b and Figure 3), wherein the spread of ER is indeed attributed to the destructive catalytic function of this ribozyme (Figure 4-*P_BER_* and *T_ER_*), and its encoding in a circular genome was shown to be crucial for this spread (Figure 2c).

Somewhat surprisingly, it was found that, though the ribozyme itself, as a linear molecule, may be attacked by other ER molecules, the spread is barely affected. For example, the spread of ER is robust against the downward adjustment (even to 0) of the assumed probability that a ribozyme is protected (Figure 4-*P_RP_* and Figure S3a; note that this probability may have a little influence on the quantity of linear ER—comparing Figure S3b with Figure S3c). The reason should be that the degradation of linear ER would also generate building blocks for further synthesis of its own species. As a conclusion, we highlight that a circular genome is critical (Figure 2c) and sufficient to sustain ER proliferation.

In our study, we mimicked the emergence of the first circular ER molecules by inoculating them periodically and successfully demonstrated their proliferation (Figure 2d). However, we found it challenging to model their de novo emergence: the parameter regimes favorable to their spread appear unfavorable for the initial formation of the first ER molecules. We suspect that reproducing this scenario would require integrating an independent subsystem with a high *P_RL_* (probability of random ligation of nucleotides and RNA) and a low *P_MV_* (probability of nucleotide and RNA movement), coupled to our core model—analogous to a pond with a mineral-surface-lined bottom (see references [71,72,73,74] for the proposed catalytic roles of mineral surfaces within the RNA world). This extension would further increase the complexity of our already intricate model and introduce an extra computational burden. Fundamentally, under Darwinian evolutionary principles, the key property of any RNA species is actually its capacity to spread once it arises. For this reason, we opted not to implement this additional subsystem. When we checked the RNA chain length and topology distribution regarding the destructive ribozyme (ER or ER13) and constructive ribozyme (REP or NR) spreading systems (Figure 5 and Figure S5), interestingly, we found that the former seems more robust against parasites. In particular, there are far fewer linear parasitic RNAs in such spreading systems. The phenomenon is understandable because the destructive ribozyme tends to destroy them. This is significant because, even for an initial single-gene circular genome containing non-coding sequences, frequent “false breaking or synthesizing” events can still occur (e.g., breaks arising within the ribozyme-characteristic domain; see Figure 1b), thereby generating quite a lot of linear parasitic RNAs. Thus, this also supports the idea that such destructive ribozymes likely emerged at the very beginning.

Furthermore, it has been demonstrated that these constructive ribozymes can emerge following the spread of ER (Figure 6). Specifically, an ER-REP two-gene circular genome is capable of propagation (Figure 6a), which can be attributed to the functions of the two ribozymes (Figure 6b). The REP gene was then shown to arise naturally via mutation from a non-coding sequence within an ER genome, thereby driving the spread of the ER-REP genome (Figure 6c and Figure 7). Notably, REP-catalyzed ligation is the chemical reverse of ER-mediated cleavage, making them directly related in catalytic chemistry. For instance, many small self-cleaving ribozymes catalyze RNA ligation to varying degrees [56,57,58]. In fact, the earliest engineering efforts to construct an REP were based on the group I self-splicing intron [27], which mediates cleavage and ligation reactions simultaneously. Additionally, the recent identification of a short REP candidate (45 nt in length) [41] implies that the emergence of an REP in an RNA pool dominated by an ER (e.g., 20 nt in length) may be feasible.

Remarkably, an ER-REP-NR three-gene circular genome was also found capable of spreading in the system (Figure 6d). Indeed, some previous studies by our group and others suggest that the cooperation of more than two types of ribozymes—with unlinked genes—would be difficult in the naked stage of the RNA world [51,52,87]. Interestingly, the observation here shows that at least three types of ribozymes may co-spread in a purely spatial system (that is, without membrane boundaries of protocells), provided their genes are linked together. Notably, we do not propose that REP arose after ER, with NR appearing subsequently; instead, we argue that constructive ribozymes may have emerged after ER, enabling the formation of circular genomes carrying multiple genes.

As mentioned in the Introduction, ribozymes should be adept at cleaving RNA chains, and thus such functional ribozymes are still widely distributed in the protein-dominated modern living world [63,64]. However, to date, no exonuclease ribozyme (ER) has been identified. In other words, there is no biochemical evidence for ribozymes capable of cleaving RNA termini, either found naturally in modern organisms or synthesized via RNA engineering. Notably, however, a strictly processive exonuclease is actually not required—we demonstrated that a ribozyme capable of randomly cleaving an RNA substrate by 1, 2, or 3 nt at its chain end (i.e., ER13) can also propagate within the system (Figure S6). Remarkably, experimental work has shown that RNase P—a well-characterized nuclease with an RNA catalytic core—may catalyze such random cleavage when acting in trans on short RNA substrates [75,76,77], a behavior partially analogous to the function of the ER13 modeled in this work. Indeed, the catalytic RNA of RNase P is itself excessively large (300–400 nt), but interestingly, it has been revealed that small RNAs corresponding to a domain of this ribozyme (about 30 nt in length) may also perform its cleavage function, albeit with substantially reduced efficiency [77,88]. It seems not impossible to identify a small ER (e.g., no longer than 20 nt, not necessarily with very high efficiency) in the laboratory, potentially via in vitro evolution. The successful identification of such a ribozyme in reality would provide us with a glimpse of the earliest scenario of Darwinian evolution proposed here. In this respect, while the catalytic efficiency of short ER variants, their conserved sequence/structural motifs, and suitable in vitro selection schemes constitute compelling research directions, concrete notions for these targets remain unclear at present.

In the field of life’s origins, integrating experimental and theoretical approaches is significant, as neither is sufficient on its own [11]. A recent study from our group provided a paradigmatic example in which experimental clues guided corresponding theoretical modeling [89]. Here, we present a reciprocal case in which theoretical modeling can inform targeted experimental investigation. Collectively, our findings regarding an ER encoded in a circular genome offer a new perspective on the critical early stage of the RNA world’s emergence and may expand both theoretical and experimental frameworks for understanding the origin of life.

## 4. Methods

### 4.1. The Events Occurring in the Model System

**Figure 8:**
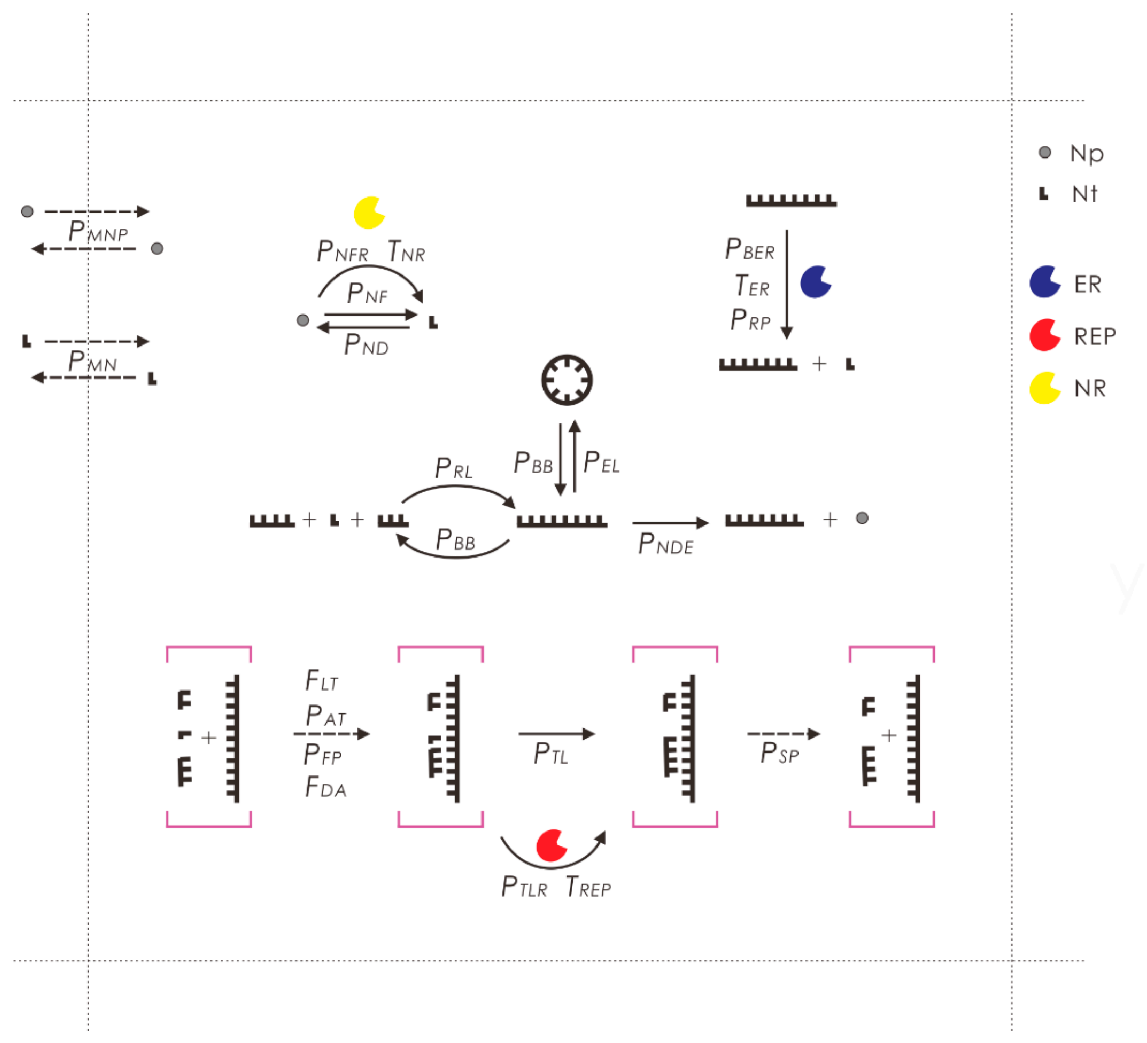
The events occurring in the model and their associated parameters. Legends: Np—nucleotide precursor; Nt—nucleotide; ER—exonuclease ribozyme; REP—RNA replicase; NR—nucleotide-synthetase ribozyme. Solid arrows denote chemical reactions, and dashed arrows represent other events. The events related to template-directed synthesis are depicted here with respect to a template segment (within purple square brackets), which may belong to either a circular RNA or a linear RNA. Note that the region shown here represents one grid room in the *N* × *N* grid of the model system. See the text for detailed explanations.

Figure 8 shows a schematic of the events in each time step (i.e., Monte Carlo step), together with their associated probabilities and factors (see Table 1 for relevant definitions). In the *N* × *N* grid system, molecules only interact with others within the same grid room. A molecule may move to an adjacent room (related probabilities: *P_MN_* for nucleotides and RNA, and *P_MNP_* for nucleotide precursors, respectively).

A nucleotide precursor may form a nucleotide (randomly A, G, C, or U) in a non-enzymatic way (*P_NF_*) or catalyzed by NR (*P_NFR_*). A nucleotide may also decay into its precursor (*P_ND_*). Nucleotides and linear RNAs may undergo random intermolecular ligation (*P_RL_*) to form longer chains. A linear RNA may undergo intramolecular end-to-end ligation (*P_EL_*) and thus transform into a circular one. An RNA molecule may attract substrates (nucleotides or oligomers) (*P_AT_*) via base-pairing with a certain error rate (*P_FP_*). Attraction of a substrate adjacent to one already positioned on the template is easier than de novo attraction (*F_DA_*)—known as the primer effect. In addition, linear RNA may act as a template less efficiently than circular RNA (*F_LT_*). Substrates aligned adjacently on the template may be ligated in a non-enzymatic way (*P_TL_*) or catalyzed by REP (*P_TLR_*), corresponding to template-directed synthesis. The substrates or the full complementary strand may separate from the template (*P_SP_*). Phosphodiester bonds within an RNA chain may break (*P_BB_*): a circular RNA may thus become linear, and a linear RNA may split into fragments. A nucleotide residue at the end of an RNA chain may decay into a nucleotide precursor (*P_NDE_*). An RNA molecule may be cleaved at its chain end by ER (*P_BER_*); however, if the molecule acts as an active ribozyme, it is protected against cleavage to a certain degree due to its folding (*P_RP_*). ER, REP and NR can catalyze their respective reactions multiple times per Monte Carlo step (*T_ER_*, *T_REP_* and *T_NR_*, respectively).

Notably, similar to our previous modeling work on scenarios in the RNA world, the energy issue is not considered explicitly here. For instance, nucleotides and oligonucleotides are implicitly assumed to be activated; in particular, upon their release from RNA degradation, they are assumed to be immediately reactivated so that they can be reused in subsequent RNA synthesis. In fact, such in situ activation has been shown to be feasible in laboratory experiments [90,91,92]. In reality, the energy source for primordial life may have involved chemical energy in prebiotic habitats, such as submarine hydrothermal vents [93,94,95] or terrestrial hydrothermal fields [96,97], as previously supposed. Put differently, all building blocks—both monomers and oligomers—once generated via synthesis or RNA degradation, might have indeed undergone immediate reactivation within these energy-abundant habitats. Introducing independent sub-modules to explicitly track reactivation and cyclic synthesis would make our already complex model far more intricate and computationally expensive. Based on the above considerations, we omit explicit modeling of energy-related reactivation processes, even though this constitutes a notable simplification of prebiotic chemistry. Since substrates are assumed to be always “activated”, the RNA species in the model system compete for materials but not energy. As noted earlier, the total amount of materials in the system is limited (*T_NPB_*). Certainly, in actual Darwinian evolution, competition for materials and energy is both possible.

### 4.2. The Setting of Parameters

The probabilities and factors concerning the events in the system should be set in accordance with certain rules. Reactions catalyzed by ribozymes should be much more efficient than the corresponding non-enzymatic reactions, so *P_BER_* ≫ *P_BB_*, *P_TLR_* ≫ *P_TL_* and *P_NFR_* ≫ *P_NF_*. Template-directed ligation should be apparently more efficient than intermolecular random ligation, so *P_TL_* ≫ *P_RL_*. Intramolecular end-to-end ligation of a linear RNA should be easier than intermolecular random ligation, so *P_EL_* > *P_RL_*. Here, nucleotide residues within the chain are assumed to be unable to decay, as they should be protected, whereas those at the end of the chain (which are only partially protected”) decay at a rate lower than that of free nucleotides; that is, *P*_NDE_ < *P*_ND_. Template-directed synthesis without the aid of a protease could not have very high fidelity, so *P_FP_* should not be set to a very small value. Other considerations may include: *P_BB_* may be higher than *P_RL_*, but lower than *P_NDE_*; *P_MNP_* should be higher than *P_MN_*; and *P_NF_* should not exceed *P_ND_*.

Considering the computational intensity, the total materials in the system (*T_NPB_*) are assumed to be obviously smaller in scale than the potential corresponding situation in reality; similarly, the characteristic RNA sequences for ER, REP or NR assumed here (8 nt) are likely to be much shorter than the corresponding ribozymes in reality. However, such simplifications are not considered to conflict with the fundamental mechanisms that the modeling is intended to reflect. Notably, the characteristic 8 nt sequences used here merely form an abstract domain representing the sequence-dependent specificity of ribozymes within our framework (to account for the sequence–function linkage central to Darwinian evolution). Even the relative lengths of these abstract units do not reflect real prebiotic conditions: as we outline in the Introduction, constructive ribozymes are expected to be longer than destructive ribozymes in the early RNA world.

Importantly, exploring parameter values that “support hypothetical scenarios” is both sensible and valuable in theoretical modeling of the origin of life, particularly given our highly limited knowledge of prebiotic environments and chemistry [98]. In fact, the evolutionary process during the origin of life is distinguished by a trend from simplicity to complexity—a highly rare phenomenon in nature. Therefore, any evolutionary scenario moving in this direction, if supported by modeling, deserves our attention: we may even deduce relevant prebiotic conditions from the “favorable parameter values” underlying that scenario. This type of modeling can be referred to as “reverse modeling” [98].

In the present study, bearing the rules mentioned above in mind and considering computational intensity, most parameter values were explored based on our experience from previous modeling studies on the naked RNA world [44,50,65,69,70], with particular reference to Ref. [65], which addresses circular RNAs. As mentioned in Results, the parameter exploration turned out to be quite successful and well supported the hypothetical scenario proposed herein. The default values listed in Table 1 were adopted for the case studies illustrating our findings; these choices are certainly not strictly required—in fact, the modeling results were indicated to be robust to moderate adjustments of most parameters (see Figure 4 and Figure S4).

### 4.3. Some Detailed Assumptions in Consideration of Relevant Mechanisms

A linear RNA containing the characteristic sequence of a ribozyme can function as the ribozyme only if its length is less than 1.5 times that of the characteristic sequence, because excess extraneous sequences may disrupt proper folding of the catalytic core. Therefore, for an 11 nt circular single-gene genome containing a 3 nt non-coding sequence, a linear product (generated by chain breaking or “template-directed synthesis”; see Figure 1) that includes the ribozyme’s characteristic sequence should always be an active ribozyme (e.g., the cases shown in Figure 2 and Figure S2). However, for a 16 nt circular two-gene genome or a 24 nt circular three-gene genome, a linear product that includes one ribozyme’s characteristic sequence is not always an active ribozyme (e.g., the cases shown in Figure 6)—although an overly long product may become a functional ribozyme after “appropriate” chain breaking, chain-end decay, or cleavage by ER.

An RNA template may attract a substrate (nucleotide or oligomer) at any “empty” site, provided that the substrate is no longer than the empty site. The attraction aided by a preceding substrate (acting as a “primer”) on the template is easier than de novo attraction by a factor of *F_DA_* (*F_DA_* > 1). A linear template has lower templating efficiency than a circular template by a factor of *F_LT_* (*F_LT_* < 1). That is, for primer-aided attraction on a circular template, the corresponding probability is *P_AT_*; for de novo attraction on a circular template, the probability is *P_AT_*/*F_DA_*; for primer-aided attraction on a linear template, the probability is *P_AT_* × *F_LT_*; and for de novo attraction on a linear template, the probability is *P_AT_* × *F_LT_*/*F_DA_*.

The probability of strand separation in a duplex RNA is assumed to be *P_SP_ ^r^*, where *r* = *n*^1/2^ and *n* is the number of base pairs in the duplex. When *n* = 1, the probability equals *P_SP_*. As *n* increases, the probability decreases (since *P_SP_*, as a probability, lies between 0 and 1). In other words, strand separation becomes increasingly difficult for longer duplexes with more base pairs. The exponent 1/2 accounts for the fact that self-folding of the individual single strands may promote the dissociation of the duplex.

For the chain breaking of RNA molecules, when cleavage occurs in a single-stranded region, the rate is *P_BB_*. When cleavage occurs within a double-stranded region, the two parallel bonds may break simultaneously, with a probability of *P_BB_*^3/2^. The use of the exponent 3/2, rather than 2, accounts for the synergistic effect of the two cleavage events.

Nucleotide residues at the ends of a linear RNA chain (either the 3′- or 5′-end) may undergo decay. Following similar reasoning to the RNA chain breaking described above, when the chain end is in a single-stranded state, the decay rate is *P_NDE_*; when the chain end is in a double-stranded state, the two paired nucleotide residues may decay simultaneously, with a probability of *P_NDE_*^3/2^.

A linear RNA may be cleaved at its 5′-end (single-stranded) by the ER with a probability of *P_BER_*; if the RNA is an active ribozyme, the probability becomes *P_BER_* × *P_RP_*, reflecting its resistance to cleavage due to its functional conformation. Notably, in the model, the ER represents a 5′-end nuclease—in principle, however, a 3′-end nuclease is fully equivalent with respect to its capability to spread in the system.

The probability of an RNA molecule moving is assumed to be *P*_MN_/*m*^1/2^, where *m* is the mass of the RNA relative to that of a nucleotide. This assumption accounts for the effect of molecular size on molecular motion. The square-root dependence is adopted here according to the Zimm model, which describes the diffusion coefficient of polymer molecules in solution [99].

(Note: The C-language source codes for the simulation program are available on GitHub, as stated in the Data Availability Statement. In addition to enabling independent reproduction of our simulation results, the codes contain model implementation details and can facilitate readers’ in-depth understanding of the simulation mechanism.)

## Figures and Tables

**Table 1:** Parameters in the model.

**Probabilities**	**Descriptions**	**Default Values**
*P_AT_*	An RNA template attracting a substrate (nucleotide or oligomer)	0.5
*P_BB_*	A phosphodiester bond breaking within an RNA chain	1 × 10^−6^
*P_BER_*	A phosphodiester bond breaking catalyzed by ER	0.9
*P_EL_*	The end-to-end ligation of an RNA chain (circularization)	5 × 10^−6^
*P_FP_*	The false base-pairing when an RNA template attracts a substrate	0.001
*P_MN_*	The movement of nucleotides	0.005
*P_MNP_*	The movement of nucleotide precursors	0.01
*P_ND_*	A nucleotide decaying into its precursor	0.05
*P_NDE_*	A nucleotide residue decaying at RNA’s chain end	0.001
*P_NF_*	A nucleotide forming from its precursor (non-enzymatic)	0.05
*P_NFR_*	A nucleotide forming from its precursor catalyzed by NR	0.9
*P_RL_*	The random ligation of nucleotides and RNAs	1 × 10^−7^
*P_RP_*	A ribozyme being protected (from the cleaving of ER) due to its folding	0.5
*P_SP_*	The separation of a base pair	0.5
*P_TL_*	The template-directed ligation (non-enzymatic)	0.05
*P_TLR_*	The template-directed ligation catalyzed by REP	0.9
**Others**	**Descriptions**	**Default Values**
*N*	The system is defined as an N × N grid	30
*T_NPB_*	Total nucleotide precursors introduced in the beginning	1 × 10^5^
*T_ER_*	The times for an ER to function in a time step	10
*T_REP_*	The times for an REP to function in a time step	10
*T_NR_*	The times for an NR to function in a time step	10
*F_DA_*	The factor concerning the de novo attraction of a substrate	5
*F_LT_*	The factor for a linear RNA acting as a template	0.5
*CS_ER_*	The characteristic sequence of ER	ACGAACUG
*CS_REP_*	The characteristic sequence of REP	GAGUCUCU
*CS_NR_*	The characteristic sequence of NR	CUGCAUCA
*CS_CT_*	The characteristic sequence of the control RNA	GUGAUCCA

**Notes:** The probabilities are listed in alphabetical order. The simulation cases in this study adopted the default parameter values unless otherwise explicitly stated. Detailed explanations of the parameters and guidelines for their configuration are provided in Section 4.

## Data Availability

Source codes of the simulation program can be accessed at https://github.com/mwt2001gh/er (accessed on 19 June 2026), including representative versions corresponding to the cases shown in Figure 2b,c and Figure 6c,d.

## Supplementary Materials

The following supporting information can be downloaded at https://www.mdpi.com/article/10.3390/life16081287/s1, as a combined PDF file, containing Figures S1–S6, Table S1, and Box S1.
