# Supplementary material for: Exonuclease Ribozyme Encoded in a Circular Genome: On the Initiation of the RNA World
Source: Life (Basel). 2026 Aug 4;16(8):1287. doi: 10.3390/life16081287 (PMC13514443; doi:10.3390/life16081287)
Supplement: Supplementary file 1 [file life-16-01287-s001.zip › life-4416976-supplementary.pdf]

Supporting Information for:

Exonuclease ribozyme encoded in a circular genome: On the initiation of the RNA world

Minglun Liang[1], Chunwu Yu[2], Hongyu Jiang[1], Wentao Ma[1]*

1. Hubei Key Laboratory of Cell Homeostasis, College of Life Sciences, Wuhan University, Wuhan 430072, China

2. College of Computer Sciences, Wuhan University, Wuhan 430072, China

* Correspondence to:

Wentao Ma

College of Life Sciences,

Wuhan University,

Wuhan 430072

China

Email: mwt@whu.edu.cn

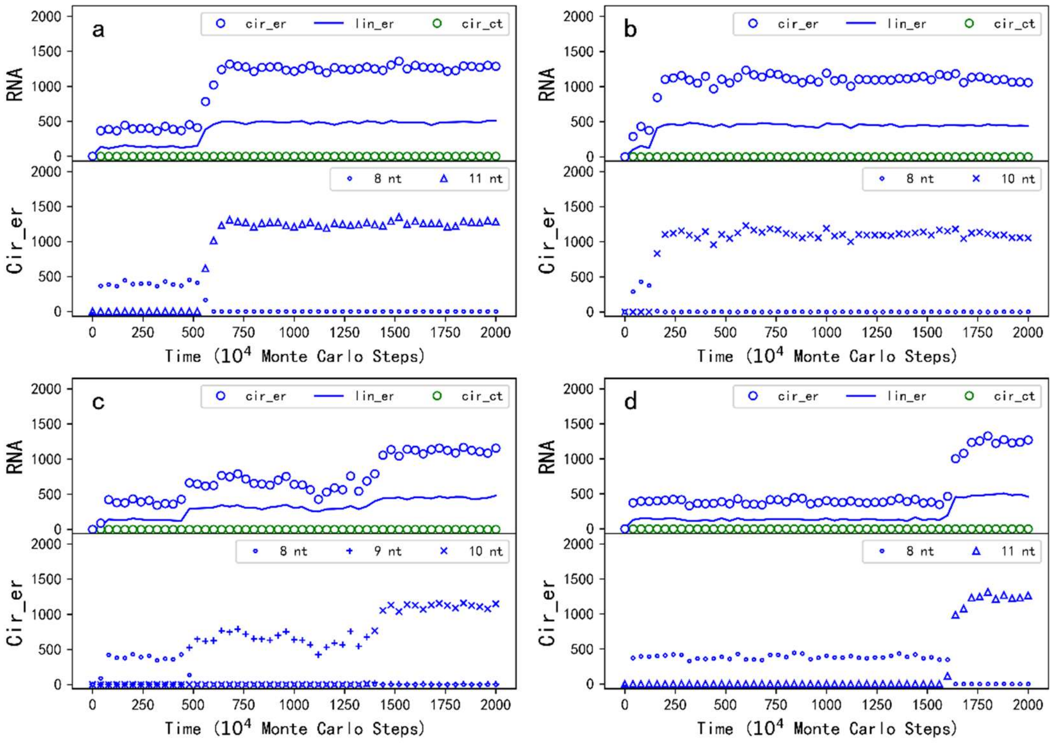

Figure S1. Emergence of non-coding sequences in circular ER genomes. For each subfigure, the upper panel follows the format of Figure 2 (with the complementary sequences omitted); the lower panel tracks the length evolution of the circular ER genome in the same case: for example, “8 nt” denotes a genome containing only the ER characteristic sequence (see Table 1), while “11 nt” indicates a genome containing the ER sequence plus a 3-nt non-coding fragment. Case (**a**) corresponds to Figure 2a; cases (**b**), (**c**) and (**d**) show simulations performed under identical conditions but with different random seeds.

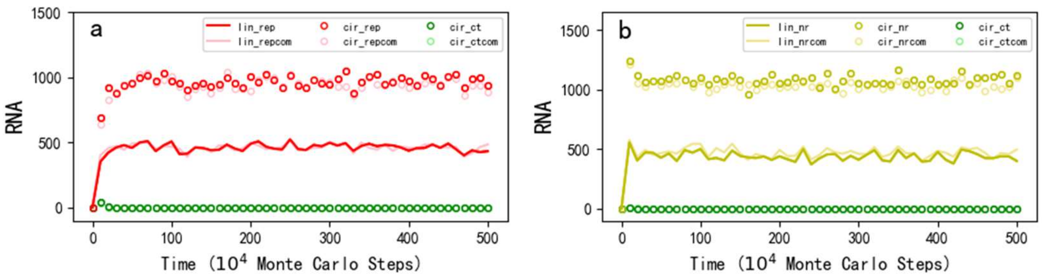

Figure S2. The spread of constructive ribozymes encoded in circular genomes. The subfigures show cases equivalent to the one presented in Figure 2b, except that the functional ribozymes are REP (**a**) and NR (**b**), respectively. In the legends, “rep” refers to REP and “nr” refers to NR. For case (*b*), P_{NF}=0.001, P_{TL}=0.1, and F_{LT}=0.3.

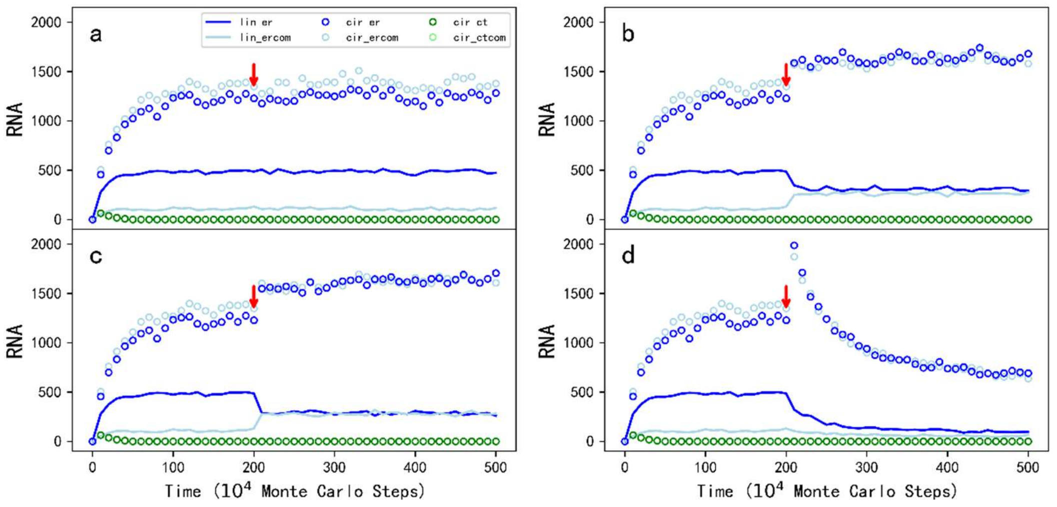

Figure S3. Mechanistic exploration of the numerical asymmetry between ER and its complementary sequence. Based on the case shown in Figure 2b, at step 2×10^6 (the red arrow): (**a**) P_{RP} is adjusted from its default value of 0.5 to 0; (**b**) linear RNAs containing the complementary sequence of ER acquire the cleavage function of ER; (**c**) both modifications described in *(a)* and (*b)* are implemented; (**d**) F_{LT} is adjusted from its default value of 0.5 to 0. The legends in *(a)* apply to all subfigures.

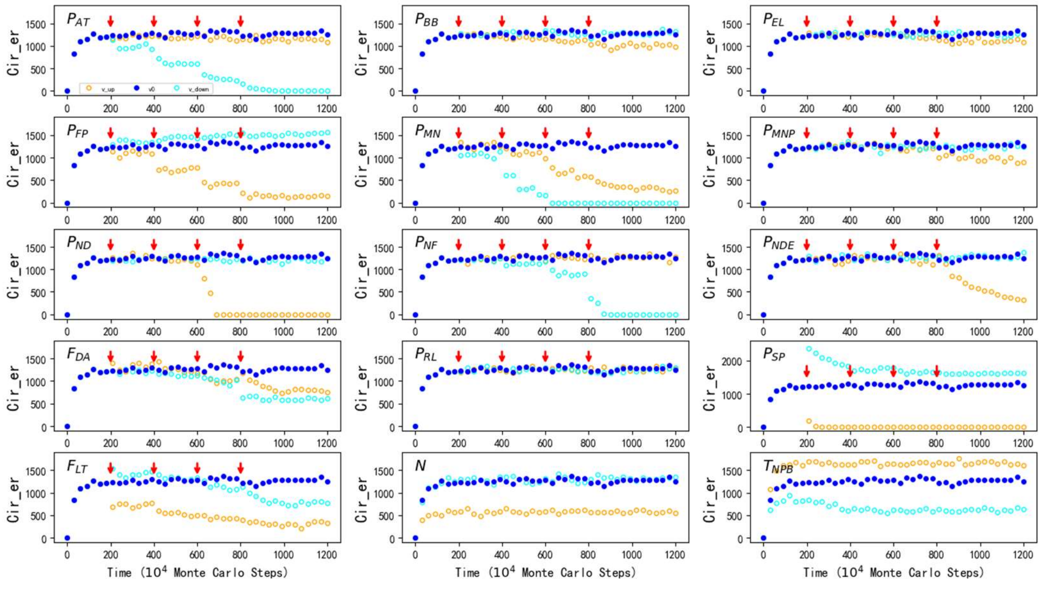

Figure S4. The influence of other parameters (in addition to those shown in Figure 4) on the spread of ER. The representations are the same as those in Figure 4. The parameter adjustments are made using a general scaling approach—when turning a parameter up or down, a scaling factor of 2 or 2.5 is typically adopted, except in cases where this would lead to unphysical values (e.g., a probability exceeding 1). A total of four critical change points (red arrows) are included to cover a broader range of parameter values. The values adopted at

these change points are listed in Table S1. For N and T_{NPB}, which are unsuitable for mid-run adjustment, non-default values are used from the very beginning—refer to the footnotes of Table S1. See Box S1 for comments on their effects.

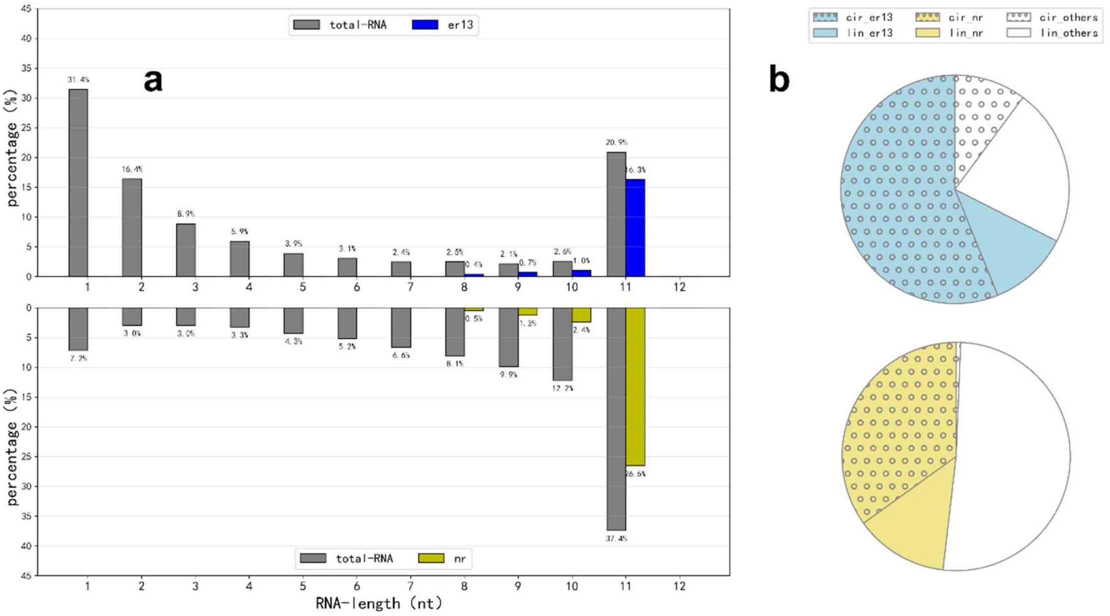

Figure S5. RNA distribution of the ER13-spreading and NR-spreading systems. The charts are presented in the same way as those in Figure 5, except that the states of two other systems (ER13 and NR) are shown instead (see Figure S6a and Figure S2b for their respective evolutionary dynamics).

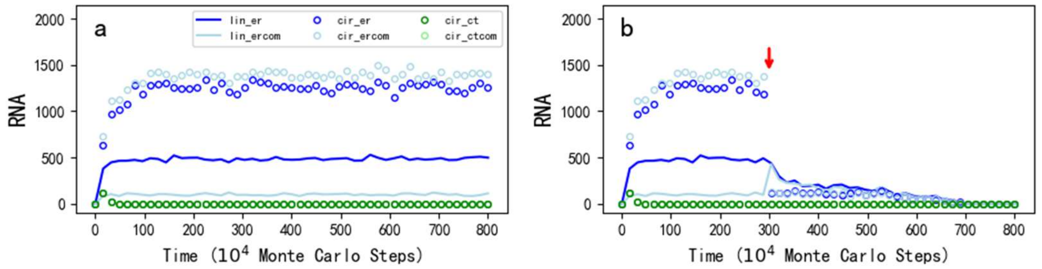

Figure S6. The spread of ER13 encoded in a circular genome. (**a**) This subfigure is explained in the same way as Figure 2b, except that ER13 (a variant of ER, see the text for explanation) is modeled here. (**b**) Based on the case shown in (*a*), P_{BER} is adjusted to 0 (i.e., the catalytic ability of this ribozyme is turned off) at step 3×10^6. The legends in *(a)* also apply to (*b*).

Table S1. Values adopted for the parameter analysis presented in Figure S4.

v_u4	v_u3	v_u2	v_u1	**v0**	v_d1	v_d2	v_d3	v_d4
0.98	0.95	0.9	0.8	$\boldsymbol{P_{AT}}$**=0.5**	0.2	0.1	0.05	0.02
2×10^{-5}	1×10^{-5}	5×10^{-6}	2×10^{-6}	$\boldsymbol{P_{BB}=1\times10^{-6}}$	5×10^{-7}	2×10^{-7}	1×10^{-7}	5×10^{-8}
1×10^{-4}	5×10^{-5}	2×10^{-5}	1×10^{-5}	$\boldsymbol{P_{EL}=5\times10^{-6}}$	2×10^{-6}	1×10^{-6}	5×10^{-7}	2×10^{-7}
0.02	0.01	0.005	0.002	$\boldsymbol{P_{FP}}$**=0.001**	5×10^{-4}	2×10^{-4}	1×10^{-4}	5×10^{-5}
0.1	0.05	0.02	0.01	$\boldsymbol{P_{MN}}$**=0.005**	0.002	0.001	5×10^{-4}	2×10^{-4}
0.2	0.1	0.05	0.02	$\boldsymbol{P_{MNP}}$**=0.01**	0.005	0.002	0.001	5×10^{-4}
0.9	0.5	0.2	0.1	$\boldsymbol{P_{ND}}$**=0.05**	0.02	0.01	0.005	0.002
0.9	0.5	0.2	0.1	$\boldsymbol{P_{NF}}$**=0.05**	0.02	0.01	0.005	0.002
0.02	0.01	0.005	0.002	$\boldsymbol{P_{NDE}}$**=0.001**	5×10^{-4}	2×10^{-4}	1×10^{-4}	5×10^{-5}
2×10^{-6}	1×10^{-6}	5×10^{-7}	2×10^{-7}	$\boldsymbol{P_{RL}=1\times10^{-7}}$	5×10^{-8}	2×10^{-8}	1×10^{-8}	5×10^{-9}
0.98	0.95	0.9	0.8	$\boldsymbol{P_{SP}}$**=0.5**	0.2	0.1	0.05	0.02
100	50	20	10	$\boldsymbol{F_{DA}}$**=5**	4	3	2	1
0.98	0.95	0.9	0.8	$\boldsymbol{F_{LT}}$**=0.5**	0.2	0.1	0.05	0.02

Notes: "v0" indicates the default value; "v_u1", "v_u2", "v_u3" and "v_u4" represent the values adjusted at the four sequential change points (marked by red arrows in the subfigures) for the parameter-turning-up case, while "v_d1", "v_d2", "v_d3" and "v_d4" correspond to the values adjusted at the four change points for the parameter-turning-down case. Adjustments to N and T_{NPB} are implemented from the beginning: for N, with a default value of 30, the turning-up case adopts 40 and the turning-down case adopts 20; for T_{NPB}, with a default value of 1×10^5, the turning-up case adopts 1.2×10^5 and the turning-down case adopts 8×10^4.

Box S1. On the influence of parameters (for Figure S4)

We briefly comment below on the cases where parameter changes lead to obvious effects. A low probability for an RNA template to attract substrates (P_{AT}) impedes the template-directed replication of ER. A high error rate in the replication of RNA (P_{FP}) is disadvantageous, as it weakens the heritability of ER. A low RNA movement rate (P_{MN}) hinders ER from accessing materials across the system, whereas an excessively high movement rate prevents the ER genome from benefiting sufficiently from its encoded ribozyme. An overly high probability of nucleotide decay (P_{ND}) causes a scarcity of RNA building blocks, thereby suppressing ER replication. Likewise, an excessively low probability of nucleotide formation (P_{NF}) also leads to nucleotide shortage. An excessively high probability of RNA end decay (P_{NDE}) may shorten the lifespan of ER to the point that the ribozyme cannot be stably maintained in the system via replication. An excessively high value of the primer-effect factor (F_{DA}; see Methods) means primers would be difficult to attach to the template, whereas a low value of this factor weakens the superiority of copying extension relative to initiation—consequently limiting the synthesis of longer strands. Both scenarios impair ER replication. A high probability of base-pair separation (P_{SP}) causes substrates to detach from the template prior to ligation during template-directed synthesis, which is detrimental to ER replication (notably, ER spread is suppressed at the first upward turning point, indicating high sensitivity to the increase of this parameter). Conversely, a moderately lower P_{SP} value can be beneficial; however, this benefit is quickly counteracted by impaired strand separation during RNA replication. A higher factor characterizing the template efficiency of linear RNAs (F_{LT}) is unfavorable, as linear RNAs compete with the circular genome for building blocks. Nevertheless, linear RNAs also cooperate with the circular genome to a certain extent (see Figure S3d and the main text for details), so an excessively low F_{LT} value is also detrimental. A larger spatial scale (N) weakens system density, reducing the efficiency of molecular interactions and reactions. A larger initial input of raw materials (T_{NPB}) increases systemic material availability and thereby facilitates ER spread; conversely, a smaller T_{NPB} restricts resource supply and inhibits ER spread.
